# Editorial: The contributions of women to respiratory physiology and pathophysiology

**DOI:** 10.3389/fphys.2023.1204020

**Published:** 2023-04-25

**Authors:** Kimberley C. W. Wang, Rachel E. Foong

**Affiliations:** ^1^ School of Human Sciences, The University of Western Australia, Crawley, WA, Australia; ^2^ Wal-yan Respiratory Centre, Telethon Kids Institute, Nedlands, WA, Australia; ^3^ School of Allied Health, Curtin University, Perth, WA, Australia

**Keywords:** gender, asthma, airway hyper responsiveness, airway remodeling, lung disease

Respiratory physiology and pathophysiology is an important field of respiratory research and can aid in the understanding of causes of symptoms in respiratory diseases. Although largely male-dominated, there are several prominent women who have made significant advances in the field, and women continue to contribute substantially to research into respiratory physiology and pathology. We are honoured to highlight the contributions some of the women have made in this field, and pleased to showcase four new research articles by female researchers in the Women in Respiratory Physiology and Pathophysiology 2022 Research Topic.

The management of asthma could have been very different if Professor Ann Woolcock (Smith, 2014) had not introduced the idea of a standardised approach and developed the world’s first guidelines for asthma management—Australian Asthma Management Guidelines, together with other researchers. She advanced the understanding of the pathophysiology of asthma, describing the differences in the shape of dose-response curves to methacholine in healthy and asthmatic subjects, and importantly, introducing the existence of the plateau on dose-response curves. Her research established the use of full-dose response curves to inhaled agonists using the pharmacological model, and these tests for airway responsiveness were adapted and are now widely used in clinical and epidemiological studies. Similarly, Janet Stocks, Professor in Respiratory Physiology, has also developed lung function testing in particular for the assessment of lung development in infants and young children, with appropriate interpretation across all age groups to ensure that any effects of disease or treatment can be distinguished from changes due to normal growth and development. Adaptation of these tests for young children have contributed to the characterisation of lung diseases such as asthma, cystic fibrosis and children born premature. Both Ann and Janet have received many well deserved national and international accolades, acknowledging their great achievements in the respiratory field.

Another important female researcher is Dirkje Postma, Professor of the Pathophysiology of Respiration, who has been pivotal in characterizing airway hyperresponsiveness (AHR), as acknowledged with numerous career awards, including a European Respiratory Society Sadoul Award, and recognised as one of the giants in chest medicine (Kerstjens, 2018). Susan Gunst, Chancellor’s Professor Emeritus, is also renowned amongst respiratory physiologists as she identified mechanisms by which airway smooth muscle cells regulate contractility and adaptation to mechanical stresses. Finally, Dr. Stephanie Shore is notable for studying the respiratory response to ozone, and the understanding of obesity as a risk factor for asthma. Both Susan and Stephanie’s distinguished contributions have been acknowledged by a Joseph R. Rodarte Award for Scientific Distinction from the American Thoracic Society Assembly on Respiratory Structure and Function.

The first paper in this special topic discusses the functional abnormality of asthma, which is AHR leading to airflow limitation. Carroll et al. first discussed the contribution of inflammation and remodelling to AHR and *in vitro*, *in vivo*, and *ex vivo* techniques used to assess AHR. The authors then reviewed various mouse models of experimental asthma highlighting that presenting % change and raw data would allow direct comparison of the degree of AHR between studies and models. Mouse models of experimental asthma could be induced by allergen, diet or infection producing different endotypes and phenotypes of asthma. Given the strong evidence for early origins of asthma, the review also discussed how fetal growth disruptions due to exposure to environmental or tobacco smoke or low maternal iron status during pregnancy can increase the risk of asthma in offspring.

The paper by Hsieh et al. reviews the heterogeneity of airway remodelling in asthma and how it may affect disease outcomes. The authors discuss how recent studies into airway remodelling have been improved with the use of computed tomography scanning and magnetic resonance imaging has enabled the 3-dimensional assessment of the lung structure *in vivo* in patients compared to previous studies using histological samples from lungs of fatal asthma patients. Furthermore, the potential of pharmacological interventions targeting airway remodelling to improve outcomes in asthmatic patients was also outlined, providing an in-depth assessment of the current state of research into airway remodelling in asthma.

Persistent pulmonary hypertension (PPHN) happens when the neonate fails to make the transition from fetal circulation to normal newborn circulation leading to other complications such as chronic lung disease or congenital diaphragmatic hernia. Hinton et al. exposed pulmonary artery smooth muscle cells (PASMC) to hypoxia to show that the cells then become a source of oxygen and nitrogen reactive species. They found that the addition of nitric oxide (NO) to hypoxic PASMC augments calcium release to thromboxane and diminishes cAMP generation by adenylyl cyclase, resulting in increased signalling for contraction. Given that inhaled NO is used as a PPHN treatment, further investigations are required to understand the efficacy of this treatment.

The final paper in the Research Topic by Garrison et al. discusses the role of pericytes in the pathophysiology of respiratory diseases. These cells, which can be found in blood vessels in the lung, have essential roles in vascular homeostasis and remodelling, as well as in tissue injury, disease, and repair. However, due to the heterogenous nature of pericytes, it has been difficult to characterise these cells, and thus their role in maintaining lung health has been underappreciated. In their review, the authors outline signalling pathways of pericytes and their interactions with other cell types. They also discuss findings from pre-clinical studies of pulmonary arterial hypertension and asthma showing the contributions of pericytes to these disorders, and their potential target for treatment.

To conclude, the Women in Respiratory Physiology and Pathophysiology 2022 Research Topic presents diverse respiratory physiology and pathophysiology research topics. Importantly, this article Research Topic has demonstrated some of the women’s research in the field, highlighting the efforts led by women researchers contributing to the respiratory physiology and pathophysiology field. This Frontiers in Physiology series represents our ongoing commitment to equity, diversity, and inclusivity in the respiratory field.

## References

[B1] Kerstjens H. A. M. (2018). Giants in chest medicine: Dirkje S. Postma, MD, PhD. Chest 153 (6), 1296–1298. 10.1016/j.chest.2018.03.002 29884249

[B2] Smith B. (2014). Ann Janet Woolcock 1937–2001. Hist. Rec. Aust. Sci. 25 (2), 313–336. 10.1071/HR14023

